# Comparison of Clinical Outcomes of Implantation of Foldable Hydrophobic Acrylic Intraocular Lens With and Without Heparin Surface Modification in Indian Diabetic Patients

**DOI:** 10.7759/cureus.56855

**Published:** 2024-03-24

**Authors:** Divya Kumari, Sriram Simakurthy, Namrata Sharma, Jeewan Titiyal, Atul Kumar, Rohan Chawla, Vijay Shankar

**Affiliations:** 1 Ophthalmology, Sri Sai Eye Hospital, Patna, IND; 2 Ophthalmology, Sankara Eye Hospital, Guntur, IND; 3 Ophthalmology, All India Institute of Medical Sciences, New Delhi, New Delhi, IND; 4 Nephrology, Nalanda Medical College and Hospital, Patna, IND; 5 Nephrology, Ruban Memorial Hospital, Patna, IND

**Keywords:** heparin surface modification, intraocular lens, cataract, central macular thickness, aqueous flare

## Abstract

Background

This study aimed to compare the clinical outcomes of a heparin surface-modified (HSM) hydrophobic acrylic foldable intraocular lens (IOL) (CT LUCIA 601PY) and non-heparin-modified hydrophobic acrylic foldable IOL (AcrySof IQ SN60WF) in diabetic patients undergoing phacoemulsification.

Methodology

This randomized, single-surgeon, double-masked controlled trial was conducted at Dr. Rajendra Prasad Centre for Ophthalmic Sciences, All India Institute of Medical Sciences, New Delhi. In this randomized controlled trial, 100 eyes of 100 diabetic patients with or without mild-to-moderate diabetic retinopathy were enrolled (HSM IOL, n = 50; non-HSM IOL, n = 50). Outcome measures were aqueous flare, visual acuity, and anterior chamber depth (ACD). These were measured preoperatively as well as one day, one week, one month, three months, six months, and one year postoperatively.

Results

The HSM IOL group had significantly lower anterior chamber aqueous flare values (photon count/ms) than the non-HSM IOL group on postoperative day one (9.97 ± 5.2 vs. 17.56 ± 11.3, p < 0.001), postoperative week one (11.47 ± 7.78 vs. 17.06 ± 9.4, p = 0.02), and postoperative month three (7.7 ± 4.1 vs. 12.5 ± 5.6, p = 0.004) of phacoemulsification. The corrected distance visual acuity (CDVA) was significantly better in the HSM IOL group on postoperative day one (uncorrected distance visual acuity: p = 0.022; CDVA; p = 0.005), but there was no significant difference at any other follow-ups. ACD was significantly longer in the HSM IOL group at all follow-ups.

Conclusions

The implantation of HSM IOL resulted in significantly lower inflammatory reactions in the early postoperative period in diabetics.

## Introduction

Patients with diabetes mellitus develop cataracts at an early age than the normal population [1]. Among diabetics, there is a disruption of the blood-aqueous barrier (BAB) even without clinically visible diabetic retinopathy [2], leading to an increased risk of postoperative complications such as cystoid macular edema (CME) and poor visual gain. Eyes with more advanced stages of diabetic retinopathy have a more severe BAB breakdown and increased aqueous flare intensity [3]. Although the technique of phacoemulsification has improved significantly, it induces significant trauma and BAB breakdown. The use of intracameral antibiotics, biocompatibility, and design of intraocular lenses (IOLs) [4,5] also influence BAB. Surgical trauma can be reduced by decreasing the duration of surgery, mechanical manipulation of the uvea, using less phacoemulsification energy, practicing microincision surgery [6,7], and choosing a clear corneal incision with less blood loss. The biocompatibility of IOLs can be improved by heparin surface modification. Various studies have compared lenses with and without surface modification in the past showing decreased inflammation with heparin surface-modified (HSM) IOLs [8,11]. A reduction in postoperative inflammation is associated with better patient comfort, reduced requirement of medications, and early visual rehabilitation. No study has compared aspheric hydrophobic acrylic foldable lenses with or without heparin surface modification in diabetics in the Indian population. Hence, we compared the clinical performance of two aspheric hydrophobic acrylic lenses with and without heparin surface modification in diabetic individuals using a laser cell flare-meter to measure flare intensity in the anterior chamber.

## Materials and methods

This prospective, randomized, double-blinded, single-surgeon clinical trial enrolled 100 eyes of 100 diabetic patients with cataracts presenting to the outpatient department of Dr. Rajendra Prasad Centre for Ophthalmic Sciences, All India Institute of Medical Sciences (AIIMS), New Delhi. Patients were recruited from January 27, 2016, over 1 year. The study was approved by the Institutional Ethics Committee, AIIMS, New Delhi (IECPG-49/27.11.2015, RT-17/27.01.2016), and the trial was registered with the Clinical Trials Registry - India (registration number: CTRI/2016/01/010484). The study adhered to the tenets of the Declaration of Helsinki.

The patients were randomized into two groups using block randomization with variable block size as the method of randomization. After obtaining written informed consent, patients underwent cataract surgery with the use of either of the lenses.

**Figure 1:**
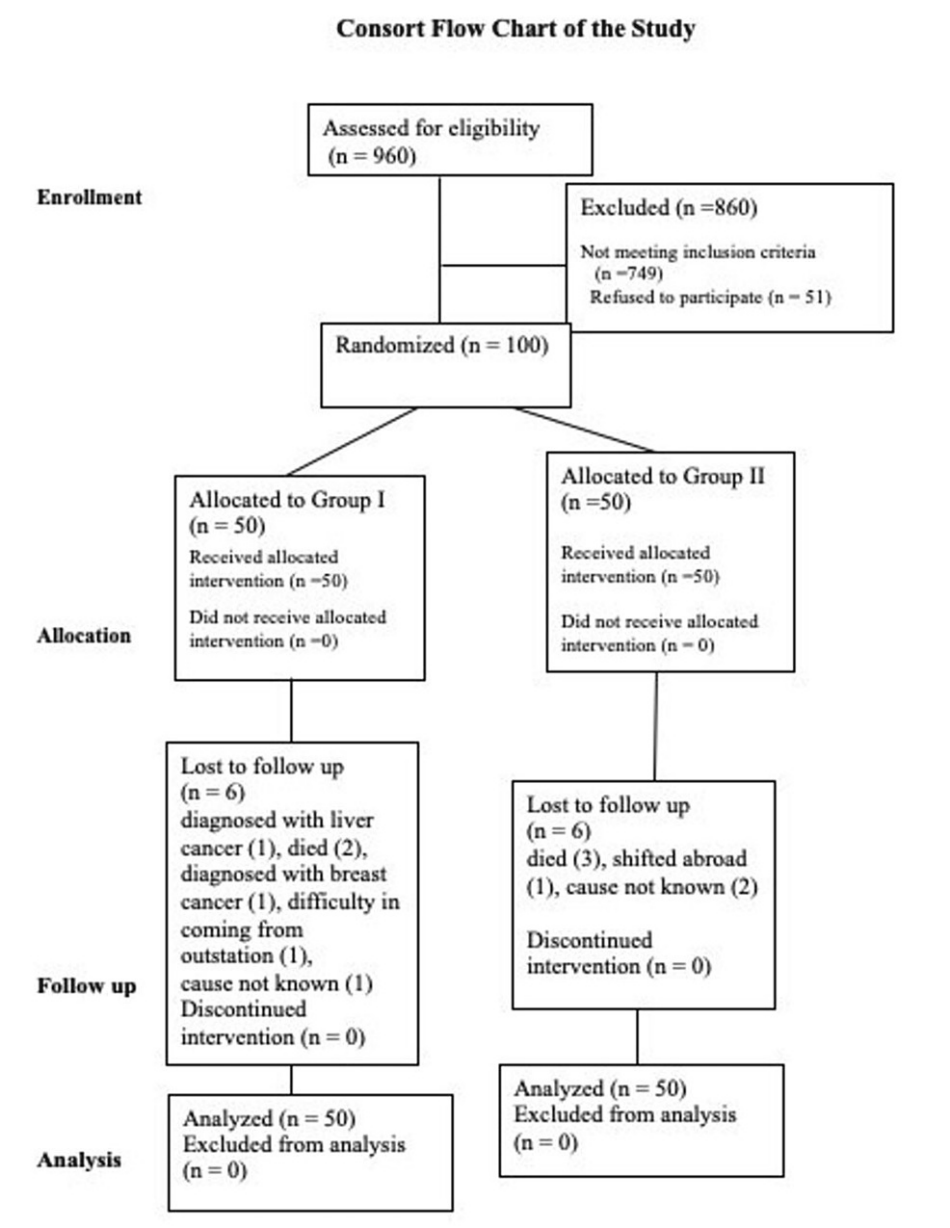
Consort flow diagram of patient selection.

The inclusion criteria were age-related cataracts in diabetic patients with no, mild, or moderate diabetic retinopathy and age of more than 45 years. Exclusion criteria were the presence of any coexistent ocular disease likely to modify visual acuity other than diabetic retinopathy (such as age-related macular degeneration, amblyopia, retinal venous occlusion), proliferative or very severe non-proliferative diabetic retinopathy, and clinically significant macular edema. Other exclusion criteria were prior intraocular surgery (trabeculectomy, vitrectomy), intraocular tumors, pseudoexfoliation syndrome, intraoperative capsule complications such as an anterior or posterior capsular tear, use of oral steroids, glaucoma, or neovascularization of the iris. The sample size was calculated to be 45 (in each group) to detect postoperative differences in aqueous cell flare. Calculations were based on confidence levels of 95% and power of 80% using PS software. Adding 10% to the sample size for non-response, errors in measurements, or loss to follow-up, the final sample size was calculated to be 50 patients in each group. The consort flowchart of the study is shown in Figure 1.

Baseline comprehensive ocular examination was performed in all cases, including slit-lamp examination to grade the type of cataract into cortical, nuclear, and subcapsular or posterior polar or hypermature. Gradings of nucleus sclerosis were based on lens coloration into pale yellow (grade I), yellow (II), brownish yellow (III), and brown including reddish and black brown (IV) [12]. Dilated fundus examination was done through an indirect ophthalmoscope, and the presence and severity of diabetic retinopathy were noted according to the Early Treatment Diabetic Retinopathy Study classification [13]. Uncorrected distance visual acuity (UDVA), corrected distance visual acuity (CDVA), aqueous flare, central macular thickness (CMT), and anterior chamber depth (ACD) were recorded in all cases and at all visits. CDVA was recorded using a Snellen visual acuity chart at a distance of 6 m. Aqueous flare was measured using KOWA FM 600. Seven readings were taken with a variation of <15% between background readings. Two extreme values were discarded, and the resulting mean and standard deviation were calculated.

Optical biometry was done using IOL Master 500, Carl-Zeiss Meditec Version 5. Where biometry was not possible on IOL Master 500 owing to the density of the cataract, an immersion scan ultrasound biometry (Alcon Ocuscan RXP Biometer) was performed.

CMT was recorded using spectral-domain optical coherence tomography (OCT) (CIRRUS HD-OCT 500). Automated Refraction was done using Zeiss Humphrey auto-refractometer 599.

Intraocular lenses

On the day of the surgery, patients were randomized to receive the HSM IOL (CT LUCIA 601PY) or non-HSM IOL AcrySof IQ SN60WF (ALCON laboratories). The IOL characteristics are summarized in Table 1.

**Table 1:** Intraocular lens characteristics. IOL = intraocular lens; HSM = heparin surface-modified

	Non-HSM IOL	HSM IOL
Optic material	Hydrophobic acrylic foldable biconvex, monofocal aspheric (-0.20) AcrySof IQ SN60WF (ALCON laboratories)	Hydrophobic acrylic foldable with heparin-coated surface monofocal, aspheric (-0.12) model CT LUCIA 601PY
Diopters (D) range	+6 to +30 D	+4 to +30 D
A constant	118.7	118.5 (recommended IOL master A constant -119.1)
Optic diameter	6.0 mm	6.0 mm
Total diameter	13 mm	13 mm
Haptic length	7 mm	7 mm
Haptic angle	0 degree	5 degrees

Preoperative care

Both groups received e/d moxifloxacin (0.5%) TDS for three days before surgery. Both groups received e/d ketorlac (0.4%) TDS for three days before surgery. On the day of the surgery, the eye to be operated on was dilated with tropicamide hydrochloride 1% eye drop three times every 10 minutes.

Surgical procedure

Standard 2.2 mm incision clear corneal temporal phacoemulsification was done using a CENTURION vision system (Alcon Laboratories, Inc.) followed by the implantation of randomized IOL.

Postoperative examinations

On day one, one week, one month, three months, six months, and one year postoperatively, all patients underwent objective aqueous flare measurement with a KOWA laser flaremeter. Manifest refraction, UDVA, CDVA, IOP by non-contact tonometer, ACD, CMT, and keratometry were determined.

Postoperative treatment comprised eyedrop prednisolone sodium phosphate 1% six times per day for one day, followed by four times per day for one month, and then tapered over one month. Eyedrop moxifloxacin 0.5% was administered three times per day for two months. Eyedrop tropicamide was administered three times a day for one week, followed by two times per day for one week, once a day for one week, and then stopped.

Statistical analysis

Statistical analysis was done using SPSS version 23.0 (IBM Corp., Armonk, NY, USA). The visual acuity values were converted to the logarithm of the minimal angle resolution units (logMAR scale) for statistical analysis. Normally distributed continuous variables were expressed as mean ± standard deviation (SD) and were compared using an independent-samples t-test. Non-normally distributed continuous variables were compared using the Mann-Whitney U test. Nominal data were compared using the chi-square test or Fisher’s exact test as appropriate. The correlation was assessed using Spearman’s correlation test. A paired-sample t-test was used to evaluate the difference from baseline within each group at one month and three months. A p-value of less than 0.05 was considered statistically significant. Generalized estimating equations were used to correct the correlation of outcomes.

## Results

The study enrolled 100 eyes of 100 patients. In total, 50 eyes of 50 patients received AcrySof IQ SN60WF, and another 50 received CT LUCIA 601PY. The mean age was 62.22 ± 8.78 years in the AcrySof group and 59.93 ± 7.6 years in the CT LUCIA group. The demographic and laboratory parameters are summarized in Table 2. The differences in age, gender, duration of diabetes mellitus, and hypertension were comparable at baseline in both groups. The preoperative investigations, such as fasting blood sugar, urine albumin, and HbA1c, were also statistically non-significant between the two groups (Table 2).

**Table 2:** Preoperative demographic and laboratory parameters.

Parameter	AcrySof IQ	CT LUCIA	P-value
Age in years (mean ± SD)	62.22 ± 8.78	59.93 ± 7.6	0.191
Gender (M: F)	24/26	32/18	0.158
Duration of diabetes (mean years ± SD)	10.13 ± 8.3	9.05 ± 6.39	0.505
Duration of hypertension (mean years ± SD)	9.41 ± 6.9	7.88 ± 6.3	0.427
Hb1Ac	7.40 ± 1.33	7.95 ± 1.79	0.115
Fasting blood sugar (mg/dL)	116.2 ± 25.58	125.3 ± 20.43	0.78
Haemoglobin (g/dL)	12.24 ± 1.28	12.69 ± 1.77	0.45
Urine albumin (mg/dL)	108.63 ± 342.66	60.70 ± 236.72	0.471
Patients on insulin	4	5	0.573
Preoperative axial length	23.24 ± 1.36	23.48 ± 0.85	0.346
Preoperative corneal refractive power (D)	44.16 ± 1.5	43.94 ± 1.4	0.504
Intraocular lens power(D)	21.54 ± 4.6	21.6 ± 2.3	0.946

The differences in preoperative axial lengths, corneal refractions, and implanted IOL power between the two groups were not statistically significant (Table 2). Both groups were similar in their type of cataract distribution (Table 3). The mean aqueous flare value of the posterior polar cataract was found to be the lowest and that of hypermature senile cataract was found to be the highest.

**Table 3:** Preoperative cataract distribution and aqueous flare.

	AcrySof IQ	CT LUCIA	P-value	Aqueous flare (mean ± SD)
Nuclear sclerosis I (±subcapsular cataract ± cortical cataract)	7	7	1.0	6.0 ± 3.0
Nuclear sclerosis II (±subcapsular cataract ± cortical cataract)	14	15	0.826	6.6 ± 4.1
Nuclear sclerosis III (±subcapsular cataract ± cortical cataract)	17	16	0.829	7.16 ± 3.2
Nuclear sclerosis IV (±subcapsular cataract ± cortical cataract)	5	7	0.538	6.7 ± 2.6
Posterior polar cataract	0	3	0.079	3.0 ± 0.635
Hypermature senile cataract	7	2	0.081	14.45 ± 7.5

Intraoperative parameters such as total ultrasound time, total fluid aspirated, cumulative dissipated energy (CDE), and total duration of surgery were comparable (Table 4). CDE was significantly related to urine albumin (Pearson correlation coefficient = 0.349, p = 0.025). The AcrySof IQ group had four patients with mild diabetic retinopathy and four patients with moderate non-proliferative diabetic retinopathy (NPDR). While the CT LUCIA group had three patients with mild diabetic retinopathy and four patients with moderate NPDR. The difference was statistically non-significant (Fisher’s exact p = 0.926).

**Table 4:** Intraoperative parameters.

Parameter	AcrySof IQ	CT LUCIA	P-value
Total ultrasound time (seconds)	51.22 ± 13.54	51.66 ± 16.22	0.951
Total fluid aspirated (mL)	52.44 ± 13.54	52.44 ± 16.22	0.999
Cumulative dissipated energy	14.02 ± 7.67	11.59 ± 6.47	0.131
Total duration of surgery (minutes)	5.19 ± 1.46	5.14 ± 1.46	0.869

Three eyes in the CT LUCIA group and two eyes in the AcrySof IOL group had intraoperative floppy iris syndrome grade I (p = 0.786). One eye in the AcrySof IOL group and none in the CT LUCIA group had a shallow anterior chamber during surgery (p = 0.309). There was no incidence of posterior capsular rupture or Descemet’s membrane detachment in either of the groups. All surgeries were sutureless.

Both groups had statistically significant visual improvements on day one, week one, month one, month three, month six, and year one postoperative follow-up (all p < 0.001) (Table 5). The mean CDVA was significantly better in the CT LUCIA group on day one postoperative. Overall, 58.5% of cases in the CT LUCIA group and 41.5% of cases in the AcrySof IQ group achieved CDVA 20/30 or better on day one. However, there was no statistically significant difference in any visual outcome parameter between the two groups at any other point in time. The mean spherical refractive equivalence in the AcrySof IQ group was -0.367 ± 0.7 and -0.236 ± 0.39 in the CT LUCIA group at one-month follow-up (p = 0.48). Thus, the postoperative refractive error in the AcrySof IQ group was more myopic.

**Table 5:** Comparison of mean corrected distance visual acuity between AcrySof IQ and CT LUCIA groups (logMAR scale).

	AcrySof IQ	CT LUCIA	P-value
Preoperative	0.87 ± 0.65	0.66 ± 0.52	0.141
Postoperative 1 day	0.176 ± 0.11	0.092 ± 0.06	0.005
Postoperative 1 week	0.11 ± 0.08	0.06 ± 0.13	0.10
Postoperative 1 month	0.10 ± 0.14	0.06 ± 0.10	0.23
Postoperative 3 month	0.10 ± 0.09	0.06 ± 0.15	0.11
Postoperative 6 month	0.11 ± 0.08	0.05 ± 0.08	0.13
Postoperative 1 year	0.11 ± 0.12	0.05 ± 0.09	0.08

There was no statistically significant difference at baseline in aqueous flare values measured with a laser cell flaremeter between the two groups in the non-operated eye (Table 6). However, the preoperative aqueous flare values in the operated eye were significantly higher in the AcrySof IQ group. The preoperative and post-operative ACD comparisons between the two groups are shown in Table 7. The CT LUCIA group had larger ACD compared to the AcrySof IQ group preoperatively but the difference was statistically non-significant. However, the postoperative ACD was significantly larger in the CT LUCIA group.

**Table 6:** Comparison of mean aqueous flare between AcrySof IQ and CT LUCIA groups.

	AcrySof IQ	CT LUCIA	P-value
Preoperative non-operated eye	7.69 ± 3.77	6.89 ± 4.22	0.405
Preoperative operated eye	9.1 ± 5.74	5.7 ± 2.5	0.001
Postoperative 1 day	17.56 ± 11.3	9.97 ± 5.2	0.000
Postoperative 1 week	17.06 ± 9.4	11.47 ± 7.78	0.02
Postoperative 1 month	14.15 ± 5.95	10.9 ± 9.1	0.125
Postoperative 3 month	12.5 ± 5.6	7.7 ± 4.1	0.004
Postoperative 6 month	7.6 ± 5.0	5.7 ± 2.0	0.45
Postoperative 1 year	7.86 ± 4.8	5.2 ± 2.2	0.204

**Table 7:** Anterior chamber depth comparison between AcrySof IQ and CT LUCIA groups.

	AcrySof IQ	CT LUCIA	P-value
Preoperative	3.09 ± 0.35	3.25 ± 0.44	0.098
Postoperative 1 day	4.33 ± 0.54	4.69 ± 0.59	0.008
Postoperative 1 week	4.29 ± 0.43	4.72 ± 0.52	0.001
Postoperative 1 month	4.23 ± 0.41	4.6 ± 0.38	0.000
Postoperative 3 month	4.22 ± 0.41	4.49 ± 0.39	0.049
Postoperative 6 month	4.24 ± 0.35	4.47 ± 0.38	0.081
Postoperative 1 year	4.22 ± 0.29	4.47 ± 0.41	0.075

A highly positive correlation was found between urine albumin and preoperative aqueous flare in the operated eye (Pearson correlation coefficient = 0.520, p = 0.001) as well as between urine albumin and non-operated eye aqueous flare values preoperatively (Pearson correlation coefficient = 0.641, p < 0.001). There was no significant difference between the baseline mean aqueous flare between operated and non-operated eyes in either of the groups. Following cataract surgery, there was a significant increase in aqueous flare in AcrySof IQ (92.96%) and CT LUCIA (74.91%) (p<0.001). CT LUCIA group had significantly lower flare values than AcrySof IQ Group on postoperative day one, postoperative week one, and postoperative month three of surgery. In the postoperative month one, there was no significant difference between the two groups. The mean flare values in the CT LUCIA group were lower than the AcrySof IQ group on all postoperative visits. There was a rise in flare in the CT LUCIA group at one week followed by a gradual decline over six months. The mean flare value was less than the preoperative flare value at six months and one year in both groups. Statistically significant differences were found between the preoperative flare value and those measured on days one, week one, month one, and month three after surgery in the AcrySof IQ group but no statistically significant differences were found between the preoperative flare value and those measured at six months and one year postoperatively. However, in the CT LUCIA group, statistically significant differences were found between the preoperative flare value and those measured on day one, week one, and month one after surgery, but no statistically significant differences were found between the preoperative flare value and those measured at three months, six months and one year after surgery. The difference in preoperative and postoperative aqueous flare values became statistically non-significant at one month in the CT LUCIA group and at three months in the AcrySof IQ group. When preoperative flare was compared according to the grade of cataract, it was found to be lowest among posterior polar cataracts and highest among the hypermature senile cataract group. The rest of the cataract types had intermediate flare values (Table 3). A highly positive correlation was found between urine albumin and preoperative non-operated eye flare (Pearson correlation coefficient = 0.641, p < 0.001), as well as between urine albumin and preoperative aqueous flare of the operated eye (Pearson correlation coefficient = 0.520, p = 0.001). Aqueous flare was positively associated with the duration of diabetes mellitus or hypertension or the age of the patient (b = 0.486, p = 0.002). There was a positive correlation between Hb1AC and non-operated eye aqueous flare (Pearson correlation coefficient = 0.267, p = 0.025). No correlation was found between HbA1c and preoperative operated eye aqueous flare and postoperative aqueous flare on day one and week one. However, there was a significant correlation between HbA1c and operated eye aqueous flare at one month (Pearson correlation coefficient = 0.434, p = 0.001) and three months (Pearson correlation coefficient = 0.403, p = 0.018). Fasting blood sugar was positively correlated with non-operated eye preoperative aqueous flare (Pearson correlation coefficient = 0.246, p = 0.043) but not with operated eye preoperative aqueous flare. On regression analysis, we found that aqueous flare was positively associated with HbA1c (b = 0.88, p = 0.015) keeping other factors such as CDE, axial length, duration of hypertension, duration of diabetes mellitus, total duration of surgery, urine albumin, fasting blood sugar and age of the patient constant. Moreover, CDE was found to be positively associated with flare (b = 0.22 and p = 0.005) keeping the other covariates constant. Urine albumin was found to be positively associated with flare (b = 0.005, p = 0.017).

## Discussion

This study was designed to evaluate the clinical effect of heparin modification in hydrophobic acrylic foldable IOL by comparing it to non-HSM hydrophobic foldable IOL in diabetic cataracts. Heparin has anti-inflammatory properties [14]. A laser cell flaremeter is the most accurate and sensitive method to measure both clinical as well as subclinical anterior chamber flare which indirectly represents BAB damage or inflammation. Our study as well as a previous study by Ursell et al. has found that anterior chamber flare increases with age [15]. Previous studies have found aqueous flare value increases with an increase in the degree of nuclear opacity [15]. Our study could not find any relationship between the degree of nuclear opacity and anterior chamber flare; however, the HMSC group had higher anterior chamber flare compared to the rest. This is probably because HMSCs are larger than normal lenses with shallower ACD, leading to increased rubbing of the cataractous lens against the iris pigment epithelium. We also noted that ACIOL had a higher aqueous flare value (24.2) than PCIOL in the bag. Urine albumin correlated significantly with preoperative aqueous flare in both eyes. This might be because albuminuria heralds the onset of systemic vasculopathy [16], is a powerful predictor of major cardiovascular events [17], and might be associated with BAB disruption as well.

This study found a significant correlation between preoperative as well as postoperative flare values until six months follow-up, similar to a previous study [15], suggesting that each eye has innate BAB function.

A previous study on NPDR patients by Gatinel et al. found the highest flare value always on postoperative day one [4], and day one mean flare was higher, both in soft acrylic (18.46 ± 11.49) and in HSM PMMA (23.65 ± 12.66) than our study (9.97 ± 5.2 in HSM hydrophobic acrylic and 17.56 ± 11.3 in non-HSM hydrophobic acrylic). This may be due to surgical technique as phacoemulsification was done through a 3.2 mm sclerocorneal incision and IOL was implanted through a 6 mm scleral incision which was larger than the 2.2 incision size of our study. Another study by Krepler et al. with sclerocorneal incisions of size 6 mm (HSM PMMA) and 4 mm (non-HSM acrylic) in diabetics had day one flare values of 28.8 ± 21.0 and 25.6 ± 15.0, respectively [18]. Smaller incision sizes are related to a lower value of aqueous flare, and laser photometry is a useful tool for objectively assessing surgical technique [19].

In our study, we found that individual flare values were not necessarily highest on the first postoperative day. In the CT LUCIA group, the aqueous flare was higher at week one follow-up than the day one and one month. This might mean that the actual effect of heparin was highest in the one-week duration or the early postoperative period. This may be because apart from surgical trauma, flare values are affected by other factors such as CME, ACD, and IOL biocompatibility. As phacoemulsification technique as well as IOL biocompatibility have improved significantly in recent times, inflammation due to these have been minimized, and this might be the reason that flare values were not always highest on a postoperative day one. However, we did find a significant negative correlation between preoperative flare and preoperative ACD (Pearson correlation coefficient = 0.282, p = 0.014). The preoperative ACD correlated significantly negatively with postoperative aqueous flare on day one also (correlation coefficient = 0.302, p = 0.015). This means that shallow ACD preoperatively corresponds with higher postoperative inflammation. A similar finding was demonstrated by Pahlitzsch et al. [20] who observed that a flat anterior chamber, low anterior chamber volume, and a narrow anterior chamber angle were parameters associated with higher intraocular inflammation after femtosecond laser-assisted cataract surgery. This might also explain the higher flare value in the AcrySof IQ group preoperative value in the operated eye.

The postoperative refractive error in the AcrySof IQ group was more myopic. Theoretically, a change in the IOL position to the anterior direction leads to myopia and vice versa [21]. We speculated that as CT LUCIA had haptic angulation of 5 degrees, it resulted in a larger displacement of IOL position posteriorly compared to AcrySof IQ and deeper ACD.

One limitation of the study is that the two IOLs have different haptic angulation which increases ACD and hence aqueous flare value by either dilution effect or by decreased rubbing of IOL over with the iris. However, no correlation was found between postoperative ACD and aqueous flare values in our study.

## Conclusions

HSM IOL had a significantly lower inflammatory reaction in the early postoperative period with faster disappearance of inflammatory signs in terms of decreased aqueous flare and early visual rehabilitation in diabetics. Long-term follow-up and larger studies with similar IOL characteristics such as haptic angulation are needed to determine the actual advantages of heparin to lower postoperative inflammatory reaction and posterior capsule opacification. Moreover, we need further studies to assess the intraocular duration of action of heparin-coated IOLs.
